# Protein Phosphorylation in Cancer: Unraveling the Signaling Pathways

**DOI:** 10.3390/biom12081036

**Published:** 2022-07-27

**Authors:** Peter Coopman

**Affiliations:** IRCM U1194, INSERM, University Montpellier, ICM, CNRS, F-34298 Montpellier, France; peter.coopman@inserm.fr; Tel.: +33-411-28-31-07

The discovery of protein kinase playing key roles in cancer formation and progression has triggered great interest and stimulated intense research on signaling pathways to develop targeted treatments, as well as to identify prognostic and predictive biomarkers. Although the majority of efforts have been focused on tyrosine kinase inhibitors (TKIs) and tyrosine kinase receptor (RTK)-targeting antibodies, efforts and interests are also being directed towards serine/threonine kinases and protein phosphatases.

Unfortunately, inhibitors often lack specificity and affect various kinases. In addition, treated tumors acquire drug resistance and relapse, requiring second-line treatments. With the advent of precision medicine, it is clear that networks are more robust biomarkers than individual proteins and genes. Drug development is moving to dynamic signaling network targeting. In the postgenomic era, post-translational modifications such as protein phosphorylation and how they affect activity or network architecture remain poorly understood. 

This Special Issue, dedicated to the unraveling of the protein phosphorylation signaling pathways in cancer, includes seven review articles and six original research papers from over 80 scientists from more than seven countries worldwide.

Two review manuscripts provide an overview on the serine/threonine protein kinases PKD and PKCθ. Zhang et al. [1] discuss the Protein Kinase D 1, 2 and 3 (PKD) family members that operate in the diacylglycerol second messenger signaling network, affecting a multitude of basic cell functions in different biological systems and disease models. The dysregulation of PKD isoform expression and activity has been detected in many human diseases. This review focuses on cancer-associated biological processes (cell proliferation, survival, apoptosis, adhesion, EMT, migration, and invasion), of which the understanding is essential for the development of safer and more effective PKD-targeted therapies.

Protein Kinase C theta (PKCθ) belongs to a novel PKC subfamily and plays a role in the immune system and in the pathology of various diseases. Nicolle et al. [2] concentrate their review on its emerging function in cancer. Its increased expression leads to aberrant cell proliferation, migration and invasion, resulting in cancer initiation and malignant progression. The recent development of PKCθ inhibitors in the context of autoimmune diseases could benefit the emergence of treatment for cancers in which PKCθ is implicated. PKCs are activated by lipids in the plasma membrane and bind to a scaffold assembled on the epidermal growth factor receptor (EGFR). Using different epitope-recognizing antibodies, in their paper, Heckman et al. [3] demonstrate that PKCℇ is found in two conformations of which the active form localizes to the endosomes, ferrying vesicles to the endocytic recycling compartment, whereas inactivation counteracts this function. The other form is soluble, present on actin-rich structures, and loosely bound to vesicles. Consequently, activated PKC is persistently trafficked with EGFR and more likely to enter the endocytic recycling compartment.

Ribonucleases (RNases) of animal, fungal, and bacterial origin have been shown to be a promising tool for the development of novel anticancer drugs. Bacterial RNase from *Bacillus pumilus* (binase) exhibits a cytotoxic effect on tumor cells possessing certain oncogenes. In their experimental contribution, Ulyanova et al. [4] aimed to identify the structural parts of the binase molecule that exert the highest cytotoxic potential. They identified a peptide that shares similarities with EGF, suggesting the peptide’s specificity for EGF-receptor-overexpressing cancer cells, indicating their potential for further development as tumor-targeting peptides.

Numerous receptor tyrosine kinases exert oncogenic functions in different types of cancer, and consequently represent clinical biomarkers and therapeutic targets. The NTRK1/TrkA receptor (neurotrophin receptor kinase 1) is increasingly considered to be a therapeutic target in oncology. In breast cancer, TrkA contributes to metastasis, but its clinico-pathological significance remains unclear. In their study, Griffin et al. [5] aimed to elucidate the immunohistochemical profile and clinical ramifications of TrkA expression in a large and pathologically diverse cohort of breast cancers. They report a significantly higher protein expression of TrkA in invasive ductal carcinomas (IDCs) as compared with invasive lobular carcinomas (ILC) and ductal carcinomas in situ (DCIS). Interestingly, TrkA was significantly increased in tumors expressing the human epidermal growth factor receptor-2 (HER2). Functionally, a pharmacological Trk inhibitor reduced cell viability through decreased phospho-Tyr490–TrkA levels and downstream phospho-Ser473–AKT activation, but did not display synergy with Herceptin. These data highlight a relationship between the TrkA and HER2 receptors and suggest that TrkA may be a novel or adjunct target for HER2-positive breast tumors.

Identification of the downstream targets of protein kinases is essential for understanding their action mechanism and the discovery of novel therapeutic targets. The RET (rearranged during transfection) transmembrane receptor protein–tyrosine kinase is highly expressed in medullary thyroid cancer. In their study, Yue et al. [6] demonstrate that GDNF (Glial Cell Line-Derived Neurotrophic Factor) stimulated RET phosphorylation and resulted in a physical interaction with CDK5 and its activation by phosphorylation. Activated CDK5 further caused STAT3 activation through Ser727 phosphorylation Moreover, they also found that GDNF treatment enhanced ERK1/2 and EGR1 transcriptional regulator activity, which is involved in p35 activation. CDK5 may thus be a promising future therapeutic candidate for human medullary thyroid cancer. 

In HPV-related cervical cancer, RCC1 (Regulator of Chromosome Condensation 1) is a necessary guanine nucleotide exchange factor (GEF) in the nucleus for Ran GTPase. It acts as a critical G1/S cell cycle regulator that undergoes post-translational modifications, including phosphorylation. In their study, Hou et al. [7] experimentally established that RCC1 was phosphorylated on Ser11 in HPV E7-expressing cells via the PI3K/AKT/mTOR pathway, thereby facilitating abrogation of the G1 checkpoint. The disruption of phosphorylation of RCC1 by Ser11 mutation induced a loss of the ability of RCC1 to facilitate G1/S transition in E7-expressing cells. This study reveals a novel function of the phosphorylation of RCC1 Ser11 in high-risk HPV E7-mediated G1/S cell cycle progression and helps to better understand the molecular basis of HPV-associated carcinogenesis.

Two review papers assessed the nitric oxide (NO) and nuclear factor kappa B (NF-κB) signaling pathways and their regulation by protein phosphorylation. 

The NO free radical has been widely investigated in various cancer types and regulates different cancer-related events, which mainly depend on phosphorylating key proteins in several signaling pathways. In their review, Liu et al. [8] concentrated on the phosphorylation of central proteins, including p38 MAPK, ERK, PI3K, STAT3, and p53, which are modified by NO and that affect cell apoptosis, proliferation, angiogenesis, and metastasis, and thus provides new insight into potential targets of cancer therapy. 

NF-κB is a ubiquitous transcription factor central to inflammation and various malignant diseases, including cancer progression. The phosphorylation of NF-κB and its regulators is one of the key post-translational modifications affecting signaling. Its dysregulation often culminates in events that induce cancer. In their review, Motolani et al. [9] discuss the regulatory role of phosphorylation in NF-κB signaling and the mechanisms through which they contribute to cancer progression. They highlight some of the known and novel NF-κB regulators, such as PRMT5 (protein arginine methyltransferase 5) and YBX1 (Y-box binding protein 1), and provide future perspectives in terms of drug development to target kinases that regulate NF-κB signaling for cancer therapeutic purposes. 

The glycogen synthase kinase-3 (GSK3) [10] exists as two paralogs, GSK3α and GSK3β, which possess both redundancy and specific functions. The upregulated activity of these proteins is linked to the development of several disorders (e.g., Alzheimer’s disease) and cancer. GSK3 paralogs phosphorylate more than 100 substrates; therefore, the simultaneous inhibition of both enzymes has detrimental effects in the long term. Although the GSK3β kinase function has usually been taken as the global GSK3 activity, a growing interest in the study of GSK3α has emerged, recognizing it as the main GSK3 paralog involved in a variety of diseases. This review summarizes the current biological evidence on the role of GSK3α in Alzheimer’s disease and various types of cancer. The authors also discuss strategies that may lead to the design of the paralog-specific inhibition of GSK3α.

Although most dysregulated protein kinases behave as oncogenes, an increasing number of reports in the literature indicate that they can, in contrast, also act as tumor suppressors, depending on the cell and tissue type and cell signaling context. This has been reported for the non-receptor spleen tyrosine kinase Syk. Pharmacological Syk inhibitors are currently being evaluated in clinical trials; therefore, it is of utmost importance to better understand the signaling pathways to explain this apparent contradictory behavior. Buffard et al. [11] reconstructed and compared the Syk signaling networks using phospho-proteomic data from breast cancer and Burkitt lymphoma cell lines, in which Syk behaves as a tumor suppressor and promoter, respectively. Comprehensive bio-informatic analyses enabled them to determine the main differences in signaling pathways, network topology, and signal propagation from Syk to its potential effectors in these different cell types. It possible to reveal difficult discernable interactions among the Syk pathways that positively and negatively affect tumor formation and progression (e.g., immunoreceptor signaling, actin cytoskeleton regulation, focal adhesion and intercellular adhesion signaling, etc.). 

Protein phosphorylation results from a fine-tuned balance between kinases and phosphatases. As for the Syk kinase [11], a dual role in cancer has also been observed for the PTPN13 protein–tyrosine phosphatase, which was reviewed by Mcheik et al. [12]. They discuss PTPN13’s implication in the FAS and oncogenic tyrosine kinase signaling pathways, as well as its post-transcriptional and epigenetic regulation. They also describe the clinical significance of PTPN13 as a prognostic marker in different cancer types as well as its impact on drug sensitivity, and present recent findings on its role in cell junction regulation that implicate PTPN13 in cell death and cell migration, two major hallmarks of tumor formation and progression.

Finally, Goguet-Rubio et al. [13] reviewed the PP2A-B55 holoenzyme regulation in cancer. PP2A is one of the major serine/threonine phosphatases which is involved in the control of a myriad of different signaling cascades. Often dysregulated in cancer, PP2A, like PTPN13, is considered to act as a tumor suppressor. In their review, the authors focused on PP2A-B55, a particular holoenzyme of the PP2A phosphatase family whose specific role in cancer development and progression has only recently been highlighted. In particular, the discovery of the Greatwall (Gwl)/Arpp19-ENSA cascade, a new pathway specifically controlling PP2A-B55 activity, has been shown to be frequently altered in cancer. This review focusses on the current knowledge about the mechanisms controlling the formation and the regulation of this phosphatase and its dysregulation in cancer.

Collectively, the contributions in this Special Issue deal with numerous contemporary aspects of several protein–tyrosine and serine/threonine kinases, as well as phosphatases that either positively or negatively affect tumor formation and progression, and cover a broad range of disciplines such as proteomics, bio-informatics, cell signaling, therapeutic peptides, and clinical biomarker immunohistochemistry. These novel and exciting findings will undoubtedly stimulate further interest in the highly competitive and rapidly evolving field of kinase signaling in cancer.
